# Executive attention training effects in children aged 4 and 6 years: improvement in the trained task greater for 6-year-olds, but far transfer greater for 4-year-olds

**DOI:** 10.3389/fpubh.2025.1499924

**Published:** 2025-05-06

**Authors:** Monika Deja, Ludmiła Zając-Lamparska, Janusz Trempała

**Affiliations:** Faculty of Psychology, Kazimierz Wielki University, Bydgoszcz, Poland

**Keywords:** executive attention, training, transfer, childhood, cognitive training

## Abstract

In recent years, research on the effectiveness of cognitive training has become increasingly popular. These studies are conducted across all age groups, including both typically developing individuals and those from clinical populations. However, their results remain inconclusive. The purpose of the present study was to verify the effectiveness of executive attention (EA) training for children in the period of middle childhood and of the transfer of the training effects onto non-trained tasks engaging working memory (WM) and fluid intelligence (Gf). The sample consisted of 180 typically developing children from two age groups: 4- and 6-year-olds. The children were divided into three research groups: the Training Group (EA training, 14 sessions), the Active Control Group (naming objects, 14 sessions), and the Passive Control Group (lack of activity). In the Training Group, the computer version of the adaptive EA training was used. WM and Gf were assessed in each group in the pre-test and post-test. The obtained data indicate the existence of asymmetry in the scope of training effects. EA training leads to a higher improvement in the correctness of performing tasks in 6-year-old children than in 4-year-old children (*F* = 11.603; *p* < 0.001; η2p = 0.167). On the other hand, the transfer effect on Gf is greater in the group of 4-year-olds compared to 6-year-olds (*F* = 4.278; *p* = 0.015; η2p = 0.047), and the scope of transfer to WM is the same in both age groups (*F* = 0.772; *p* = 0.464; η2p = 0.009). The obtained results indicate the effectiveness of executive attention training in children in these age groups. The results suggest that older children benefit more from practicing specific cognitive skills. In comparison, the far transfer mechanisms of training effects seem stronger in younger children.

## Introduction

In recent years, the discipline of psychology has seen a growing popularity of studies regarding the impact of cognitive training on human cognitive functions. The research on the effectiveness of cognitive training is conducted in all age groups—children (1, 2) and adults (3, 4). The training participants also include persons from clinical groups, e.g., children with ADHD (5) or autism (6), individuals after brain injuries (7), or people who have dementia (8). Some studies compare the training effectiveness in different age groups [e.g., (9, 10)]. The most frequent types of cognitive training include attention training (comprising various functions of attention), working memory training (11), and executive functions training. Even meta-analyses of the research results of these trainings are already available. The analysis of their results shows a clear lack of research on the effectiveness of executive attention training in young children from non-clinical groups.

Executive attention (EA) is one of the three attention functions (apart from alertness and orienting) distinguished in the neurocognitive attention model proposed by Michael Posner and Steven Petersen (12, 13). According to the authors, it is responsible for the conscious, volitional regulation of the body's thoughts, emotions, and behavioral responses, especially when experiencing and resolving cognitive conflicts. It is responsible for control processes, such as resistance to distractors or interference, inhibition of the imposing reaction, temporary suspension of actions taken, and their continuation or coordination of many forms of activity simultaneously or error detection. EA, understood in this way, is a construct narrower than executive functions (EF).

Executive functions are associated with various aspects of attention (including EA) and working memory, such as selecting and retaining information relevant to the current task, and updating it, maintaining purpose, extinguishing or inhibiting, including resistance to distraction and interference, mental flexibility, planning (including setting priorities and sequences of actions), adapting goals to changing conditions, self-regulation and self-control, as well as initiating and monitoring the course of behavior (14, 15). As components of the EF, updating, attention shifting, and inhibition stand out. Therefore, it is assumed that these concepts are not identical and that executive attention is the core or hub of the development of executive functions.

The shortage of studies on childhood-only EA training is surprising for at least two reasons. Firstly, many authors suggest that the correct functioning of EA in childhood is crucial for the subsequent development of other cognitive processes at higher levels. In fact, attention is conceived as the hub for the development of EF (13), as the key element that supports the capacity of working memory (16–18), and as the foundation for intelligence (19). Along with the development of EA, the capability of behavioral and cognitive control is being formed. Executive attention is recognized as the crucial mechanism responsible for regulating behaviors and emotions in infancy and early childhood (20, 21). Studies indicate that the executive attention development level translates into school readiness and general school achievements. Executive attention is also closely related to a child's social and emotional functioning (22). A higher level of executive attention correlates positively with the acceptance by peers (23). Furthermore, the executive attention processes constitute predictors of social adjustment and potential internalizing and externalizing disorders (21).

Secondly, studies on the effectiveness of other cognitive processes training indicate a negative correlation between the age of the training participants and its effectiveness (24, 25). Researchers explain this by referring to the more extensive brain plasticity in children (26) and their neural networks which are much less specialized (27). It is asserted that cognitive interventions at a younger age result in the functional cortical activation patterns (triggered by particular cognitive tasks) becoming more extensive but less diversified (25). This should lead to a wider transfer of the effects of cognitive training in children, i.e., to the improvement in the performance of non-trained tasks. The results of such training are probably more permanent due to the early changes in the neuronal structure caused by the training (24, 25). They can also cause the effects of training, although noticeable only to a limited extent right after the intervention, to be observed in the future in the form of easier acquisition of other, more complex abilities (25). In addition, such a form of intervention as cognitive training is indicated because, according to researchers, the improvement in the efficiency of cognitive functions observed as a result is similar to that resulting from the natural maturation and development of the nervous system. Therefore, training probably accelerates the natural development path of these abilities (28, 29), following the assumption that development is “driven” by the interaction of biological maturation and experience (30). Both arguments (EA as the core of other cognitive processes and the greater effectiveness of cognitive training in childhood) justify the attempts at improving EA in children.

In this context, this raises the question regarding the significance of the child's age for the effectiveness of attention training. The way that attention functions changes radically in the long period from infancy to adulthood. This change can generally be described as the gradual transition from bottom-up attention, depending on external stimuli, to top-down attention, directed by the individual and based on the individual's goals and intentions (31). During childhood, executive attention is particularly flexible and covers control processes (13). A rapid development of executive attention can be observed between the ages of 2 and 7 (32, 33). One may ask whether this development can be additionally stimulated through training and, if so, whether differences will be observed in the effectiveness of cognitive interventions in children from various age groups and at different stages of cognitive development.

This particular problem is also connected with the significance of the starting level of cognitive resources and the effects of training. One can find two competing views on this matter (34): the magnification view assumes that the starting level of cognitive abilities is positively correlated with the effects of training, while the compensation account postulates a contrary view, i.e., the lower this level is, the greater the improvement through training becomes. The argument for the latter view asserts that individuals whose cognitive functions are very well developed at the starting point of interventions do not have any considerable possibilities of improving these functions and quickly reach the “ceiling” (i.e., the upper limit of their capabilities). These differences can modify the scope of the transfer of the training effects onto cognitive functions (35, 36). The empirical data presented in specialist publications do not indicate unequivocally which view is accurate; there are studies supporting both the magnification effect (37, 38) and the compensation effect (9, 10, 39). The meta-analysis of research results mentioned by Titz and Karbach (40) says that the training of working memory and attention in children results in the compensation effect more frequently, and it is true in the case of children who develop normally and those who manifest learning difficulties. However, no studies have compared the effectiveness of training and the scope of transfer for children from different age groups and at varying levels of cognitive development. In addition, the negative correlation between the training effectiveness and the age of the children has been confirmed on the basis of very few studies with the participation of children at significantly different ages (24). On the other hand, some authors suggest that the effects of cognitive interventions in children, though observable, can be limited by the starting (lower) level of the structural development of the brain and cognitive functions, e.g., patterns of synaptic connections or myelination of nerve fibers (30). This would mean that the effects of training are limited due to the stage of cognitive development that children are currently at—they cannot develop certain abilities or achieve a certain level of task performance if it is based on simpler, still immature processes. This means that to train a specific cognitive function, its brain correlates must be fully developed (30, 41). It is, therefore, possible that some cognitive processes are less amenable to training, at least at a certain age, because they require some advancement in the development of the nervous system. Such a way of thinking would be consistent with the concept of development in the life span theory, according to which, although development is modifiable (plastic) at all stages of development, it has its limitations, differing depending on the period of life (42). However, it is difficult to draw precise conclusions in this regard, as few studies have compared the effects of training in children of different ages. The most frequently quoted results are the averaged results obtained by children of different years and thus are at different stages of cognitive development.

### The aim of the study

The study aimed to analyze the effects of executive attention training for children in middle childhood and to compare the baseline cognitive performance and the training effects in younger and older children, i.e., 4- and 6-year-olds. Regarding the effects of training, the following were assessed: the level of improvement in trained task engaging executive attention (EA) and the scope of the transfer to the level of performance in the non-trained tasks, engaging working memory (WM), and fluid intelligence (Gf).

## Materials and methods

### Participants

One hundred eighty children participated in the study −90 children at 4 years old (*M* = 49.09 months; *SD* = 4.02) and 90 children at 6 years old (*M* = 74.12 months; *SD* = 3.60).

The criteria for inclusion in the sample were as follows: (a) age, (b) no certificate or opinion of intellectual disability or developmental disorders of the child from a psychological and pedagogical counseling center, (c) the ability of active use of speech, (d) systematic kindergarten attendance.

The participants meeting the inclusion criteria were randomly assigned to three equivalent groups: training (*n* = 60), active control (*n* = 60), and passive control (*n* = 60). Each of these groups included children from both age groups: 4-year-olds (*n* = 30) and 6-year-olds (*n* = 30). The equivalence pertained to the following variables: (1) age, (2) sex, and (3) level of fluid intelligence (see Table 1). However, among 6-year-old children, the individual groups showed significant differences in terms of the level of task performance that measured WM and EA.

**Table 1:** Characteristics of the individual experimental groups among the 4-year-old and 6-year-old children.

	**4-year-olds**				**6-year-olds**			
	**T**	**A-C**	**P-C**		**T**	**A-C**	**P-C**	
**Sex**								
n	30	30	30		30	30	30	
Girls:Boys	16:14	15:15	16:14		15:15	16:14	16:14	
**Age in months**								
M (SD)	49.93 (4.51)	48.50 (3.56)	48.83 (3.93)	*F*_(2, 87)_ = 1.040 *p* = 0.356	74.17 (3.06)	73.20 (4.57)	75.00 (2.80)	*F*_(2, 87)_ = 1.910 *p* = 0.154
Min-Max	42–56	43–56	43–55		68–79	66–79	69–78	
**TMK**								
M (SD)	13.93 (4.27)	13.23 (2.93)	15.70 (4.65)	*F*_(2, 87)_ = 2.996 *p* = 0.055	22.23 (3.19)	20.77 (5.26)	23.17 (4.13)	*F*_(2, 87)_ = 2.398 *p* = 0.097
Min-Max	7–20	7–18	7–24		17–30	11–30	13–31	
**WM mid-level** ***n***								
M (SD)	1.27 (0.39)	1.16 (0.32)	1.19 (0.37)	*F*_(2, 87)_ = 0.836 *p* = 0.437	1.57a (0.53)	2.10a (0.71)	1.79 (0.71)	*F*_(2, 87)_ = 5.045 *p* = 0.008
Min-Max	1–2.25	1–2.25	1–2.25		1–2.5	1–3.43	1–3	
**EA mid-level in session 1**								
M (SD)	2.27 (1.55)	2.83 (1.15)	2.00 (1.36)	*F*_(2, 87)_ = 2.917 *p* = 0.059	3.37a,b (2.14)	5.53a (2.32)	5.47b (2.42)	*F*_(2, 87)_ = 8.723 *p* < 0.001
Min-Max	1–7	1–7	1–6		1–7	2–9	2–9	

T, training group; A-C, active control group; P-C, passive control group; TMK, Raven's Colored Progressive Matrices.

### Procedure

The ethics committee of the Faculty of Psychology of the Kazimierz Wielki University in Bydgoszcz, Poland, approved the present study. Children from seven kindergartens in Bydgoszcz (Poland) participated in the research with the consent of the management. All parents signed a written informed consent form for their children's participation.

The study was performed on an individual basis in kindergartens, in separate and adequately prepared rooms equipped with a table, a portable computer with a 16-inch screen, and a connected joystick with two buttons. The computer was placed in the central field of view of the child. Before the start of each procedure, the joystick was placed on the table between the child and the computer, centrally in relation to the body axis, allowing the choice of either hand to perform the task. Before the study, the children had the opportunity to familiarize themselves with the joystick.

The study procedure assumed the inclusion of three groups (in each age group): experimental, active control, and passive control. The key experimental factor was the training of executive attention.

In the pre-test, all three groups had Gf measured first and then WM. Two to three days after the pre-test, children from the training group underwent computer-assisted, adaptive EA training. The training comprised 14 sessions, 3–4 times a week, 7–8 min each.

The active control group was trained with the task of sequential naming of pictures (also 14 sessions, 3–4 times a week, 7–8 min each), which served as a placebo training to control for the confounding variables resulting from a training setting (30).

The experimenter had contact with the children from the passive control group only during the pre-test and the post-test.

The post-test took place 2–3 days after the completed training in all groups.

All measurements (with the exception of fluid intelligence) were carried out using computer applications designed in the PsychoPy 1.83 software. All displayed stimuli covered 1/8 of the screen (vector graphics).

#### Pre- and post-test tasks

##### Fluid intelligence

The measurement of intelligence was based on Raven's Colored Progressive Matrices in the Polish adaptation (Test Matryc Ravena w Wersji Kolorowej—TMK) by Jaworowska and Szustrowa (43). The intelligence indicator was the total score of the test. The reliability, measured by Cronbach's alpha coefficient, was 0,91. The measurement of fluid intelligence in the pre-test and the post-test applied the same tool due to the lack of a counterpart version in Poland. The potential impact of the repeated measurement on the results was subjected to statistical control in the performed analyses.

##### Working memory

Working memory was measured by an adaptive n-back task adapted from the n-back paradigm used by Jaeggi et al. (39, 44). The child's task was to press the key indicated in the instruction when the computer screen showed the rocket in the same spot as one position/location before (1-back), two locations before (2-back), etc. The test of working memory started with the 1-back task.

The measurement of WM based on n-back tasks comprised 40+2n stimuli at each level of difficulty, of which 12 were targets, and 28+2n were non-targets. The measurement was adaptive; if the score was above 75%, the child moved to a higher n-level; if the score was from 50 to 75%, the child remained at the same n-level; and if the score was below 50%, the child retreated to easier tasks.

Considering the emotional and motivational capabilities of children, the researcher discontinued the measurement of WM when one of the following situations occurred: (1) the child performed the tasks at the given level of difficulty two times in a row and showed no improvement, (2) the child retreated to a lower level and then went back again to the higher level, but showed no improvement. Otherwise, the measurement continued until the child completed eight levels of tasks. The WM indicator was the average n-level achieved in the pre-test.

#### Executive attention training (EA)

The training consisted of tasks at 24 difficulty levels (Table 2). Each level provided 50 stimuli displayed in a random order.

**Table 2:** The structure of training tasks in particular levels of difficulty.

**Level**	**Location of stimuli**	**Properties of stimuli**	**Response**	**Exposure time/interval of stimuli**
1	Central 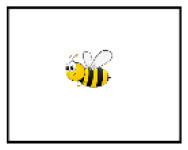	1 target (80%), 1 distractor (20%)	Response to the target with the right hand, ignoring the distractor	500/2,500 ms
2	Central	2 targets (50%/50% for the right/left hand)	Response to the targets with the right hand and the left hand	
3	Central	2 targets (40%/40% for the right/left hand) and 1 distractor (20%)	Response to the targets with the right hand and the left hand, ignoring the distractor	
4	Peripheral 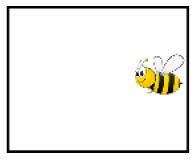 or 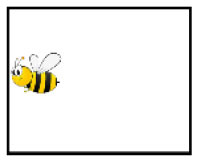	2 targets (50%/50% for the right/left hand)	Response to the targets with the right hand and the left hand, counter to the location of the stimulus	
5	Central	2 targets (50%/50% for the right/left hand)	Response to the targets with the right hand and the left hand, along the direction of the stimulus	
6	Central	2 targets (50%/50% for the right/left hand)	Response to the targets with the right hand and the left hand, counter to the direction of the stimulus	
7	Peripheral	2 targets (50%/50% for the right/left hand)	Response to the targets with the right hand and the left hand, along the direction of the stimulus	
8	Peripheral	2 targets (50%/50% for the right/left hand)	Response to the targets with the right hand and the left hand, counter to the direction of the stimulus	
9–16	As in levels 1–8			100/2,500 ms
17–24	As in levels 1–8			100/1,500 ms

The researcher discontinued the session if one of the following situations occurred: (1) the child performed the tasks at the given level of difficulty two times in a row and showed no improvement, (2) the child retreated to a lower level and then went back again to the higher level, but showed no improvement.

The child started the next training session at the level where he or she finished the previous session. However, each session used different stimuli to keep the tasks attractive to the children.

#### The image-naming task

The child's task was to name the images on the computer screen. In each session, images belonged to one semantic category (e.g., animals, vehicles) to limit the function of alternating attention (45). The child regulated the exposure time of stimuli. The response time (i.e., the time from the display of the image to the reply by the child) was not measured. The task difficulty level did not change during each session or between sessions.

### Statistical analysis

Contrast analysis was used to compare the baseline cognitive performance of 4- and 6-year-old children (without dividing them into groups in the experiment procedure).

The improvement in the training (only for children belonging to an attention training group) was evaluated using two-way repeated measurement ANOVA with age group as a between-subject predictor.

To verify the effectiveness of the training interventions, taking into account the role of age, the two-way analysis of variance was used with repeated measurements based on the following model: measurement (pre- vs. post-test) ^*^ age (4- vs. 6-y.o.) ^*^ group (Training vs. Active Control vs. Passive Control). The ANOVA analysis was supplemented with the contrast analysis to compare pre- and post-test measurements in particular subgroups (identified by age and participation in training).

## Results

### Comparison of the baseline cognitive performance in 4- and 6-year-old children

As expected, the cognitive superiority of older children over younger children was revealed in all assessed aspects. Regarding the baseline level of executive attention, we compared the average difficulty level of attention tasks reached by 4-year-old (*M* = 2.37; *SD* = 1.39) and 6-year-old children (*M* = 4.80; *SD* = 2.50) in the first measurement. These differences were found to be significant: *F*_(1, 178)_ = 65.212; *p* < 0.001, and the effect was large: $\eta_{p}^{2}$ = 0.268. We also compared the average level of “*n*” in n-back tasks achieved by 4-year-old (*M* = 1.20; *SD* = 0.36) and 6-year-old children (*M* = 1.82; *SD* = 0.69) in the first measurement of working memory. Older children's performance was again significantly higher than younger children's: *F*_(1, 178)_ = 57.514; *p* < 0.001, and the effect was large: $\eta_{p}^{2}$= 0.244. Finally, in terms of the baseline measure of fluid intelligence measured by Raven's Colored Progressive Matrices, the scores of 6-year-old children (*M* = 22.06; *SD* = 4.35) were also higher than those of 4-year-old children (*M* = 14.29; *SD* = 4.11) to a statistically significant extent: *F*_(1, 178)_ = 151.692; *p* < 0.001, and with a large effect: $\eta_{p}^{2}$ = 0.460.

### Improvement in the performance of trained tasks

First, we assessed the improvement in the training (only for children belonging to an attention training group) using two-way repeated measurement ANOVA with the age group as a between-subject predictor. The results of the first session were compared with the results of the 14th session, considering the average difficulty level of the attentional tasks achieved by the children at the indicated sessions.

During the training process, there was an improvement in the task being trained, i.e., the average level of task difficulty increased significantly (Table 3). At the same time, the degree of improvement was found to be age-dependent, as indicated by a significant interaction effect of measurement and age (Table 3). The improvement was visibly greater in 6-year-old children (pre-test: *M* = 3.37, *SD* = 2.14; post-test: *M* = 14.60; *SD* = 7.77) compared to 4-year-old children (pre-test: *M* = 2.27, *SD* = 1.55, post-test: *M* = 7.43; *SD* = 7.18). Contrast analysis, however, showed that this improvement, although less in younger children, was statistically significant in both age groups, i.e., 4-year-olds [*F*_(1, 58)_ = 16.832; *p* < 0.001] and 6-year-olds [*F*_(1, 58)_ = 79.567; *p* < 0.001].

**Table 3:** The results of two-way repeated measurement ANOVA for dependent variable: the average difficulty level of tasks engaging EA.

**Effect**	***F***	***p***	$\eta_{p}^{2}$
Measurement (pre-post)	84.795	<0.001	0.594
Age	14.391	<0.001	0.199
Measurement^*^age	11.603	<0.001	0.167

### Transfer from the training effects to working memory

Results of ANOVA indicated significant differences between the working memory task performance in the pre- and post-test (Table 4). Moreover, these differences depended on the group (training—active control—passive control), but this group-dependent pattern did not differ significantly according to age (Table 4).

**Table 4:** The results of ANOVA for dependent variable: the average n level in n-back tasks.

**Effect**	***F***	***p***	$\eta_{p}^{2}$
Measurement	37.181	<0.001	0.176
Measurement^*^group	7.011	0.001	0.075
Measurement^*^age	4.146	0.043	0.023
Measurement^*^group^*^age	0.772	0.464	0.009

The contrast analysis in the 4-year-old training group showed that the difficulty level of performed tasks increased considerably [pre-test: *M* = 1.27; *SD* = 0.39; post-test: *M* = 1.57; *SD* = 0.51; *F*_(1, 174)_ = 12.517; *p* < 0.001]. No such improvement was observed in the active control group [pre-test: *M* = 1.16; *SD* = 0.32; post-test: *M* = 1.15; *SD* = 0.33; *F*_(1, 174)_ = 0.005; *p* = 0.942] or the passive control group [pre-test: *M* = 1.19; *SD* = 0.37; post-test: *M* = 1.31; *SD* = 0.40; *F*_(1, 174)_ = 2.279; *p* = 0.133].

Six-year-old children achieved an improvement in the performance of n-back tasks in the training group [pre-test: *M* = 1.57; *SD* = 0.53; post-test: *M* = 2.05; *SD* = 0.59; *F*_(1, 174)_ = 32.903; *p* < 0.001] and in the active control group [pre-test: *M* = 2.10; *SD* = 0.71; post-test: *M* = 2.32; *SD* = 0.71; *F*_(1, 174)_ = 6.121; *p* = 0.014]. A comparison of the magnitude of improvement in these two groups indicated, on a statistical trend level, a greater improvement in the training group than in the active control group [*F*_(1, 58)_ = 3.891; *p* = 0.053)]. The passive control group showed no significant improvement [pre-test: *M* = 1.79; *SD* = 0.71; post-test: *M* = 1.93; *SD* = 0.71; *F*_(1, 174)_ = 3.068; *p* = 0.082].

### Transfer from the training effects to fluid intelligence

According to the ANOVA analysis, the post-test results in TMK differed significantly from the pre-test results (Table 5). These differences proved to depend on the group (training—active control—passive control) and are further differentiated by the age of the children (Table 5).

**Table 5:** The results of ANOVA for dependent variable: the score of the Raven's Colored Progressive Matrices (TMK).

**Effect**	***F***	***P***	$\eta_{p}^{2}$
Measurement	117.421	<0.001	0.403
Measurement^*^group	29.260	< 0.001	0.252
Measurement^*^age	0.035	0.851	<0.001
Measurement^*^group^*^age	4.278	0.015	0.047

The contrast analysis revealed that the 4-year-olds achieved an improvement in the TMK results only in the training group [pre-test: *M* = 13.93, *SD* = 4.27; post-test: *M* = 19.20, *SD* = 2.99; *F*_(1, 174)_ = 108.293; *p* < 0.001]. No significant changes were observed in the active control group [pre-test: *M* = 13.23, *SD* = 2.93; post-test: *M* = 14.20, *SD* = 3.08; *F*_(1, 174)_ = 3.648; *p* = 0.058] or in the passive control group [pre-test: *M* = 15.70, *SD* = 4.65; post-test: *M* = 16.07, *SD* = 4.74; *F*_(1, 174)_ = 0.525; *p* = 0.470].

The 6-year-olds showed an improvement in each group—the training group [pre-test: *M* = 22.23, *SD* = 3.19; post-test: *M* = 25.90, *SD* = 2.93; *F*_(1, 174)_ = 52.489; *p* < 0.001], the active control group [pre-test: *M* = 20.77, *SD* = 5.26; post-test: *M* = 22.37, *SD* = 4.84; *F*_(1, 174)_ = 9.995; *p* = 0.002] and in the passive control group [pre-test: *M* = 23.17, *SD* = 4.13; post-test: *M* = 24.73, *SD* = 4.18; *F*_(1, 174)_ = 9.583; *p* = 0.002]. However, the rate of the scores increase in the training group (*M* = 3.67; *SD* = 2.54) was significantly higher than in the active control group (*M* = 1.60; *SD* = 2.53; *p* = 0.005) and the passive control group (*M* = 1.57; *SD* = 2.13; *p* = 0.005), while there was no material difference in the rate of the scores increase between the active control group and the passive control group (*p* = 0.999).

Moreover, the comparison of training groups at different ages showed that a greater increment in the TMK results [*F*_(1, 58)_ = 4.016; *p* = 0.049; $\eta_{p}^{2}$ = 0.065] was observed in the 4-year-old age group (*M* = 5.27; *SD* = 3.56) than in the 6-year-olds (*M* = 3.67; *SD* = 2.54).

## Discussion

The purpose of the research was to analyze the effects of EA training in middle-aged children. The assessment covered the level of EA-related task performance and the scope of the transfer of the training effects onto the non-trained tasks involving WM and Gf. In addition, it was verified whether the training effects differ depending on age.

As expected, at the beginning of the study, 6-year-old children showed higher cognitive functioning than 4-year-old children in all aspects assessed, which can be explained by the level of cognitive development of children of different ages (14, 46).

The executive attention training improved the performance of trained attentional tasks, which pertained to achieving a higher level of task difficulty. The improvement was observed in both age groups. The results are analogous to those of other attention training studies with children's participation at this age. Kloo and Perner (47) recorded improvements in the trained task performance related to alternating attention. Thorell et al. (48) observed better results in trained go/no go tasks, which involve response inhibition, as well as in flanker tasks, which measured the ability to resolve cognitive conflicts. In the research by Rueda et al. (1, 49), children trained general executive attention. The children participating in the research achieved higher scores in the final measurement than in the initial measurement (1, 49).

In the training groups, the degree of improvement in the EA-related training task performance significantly differed between the pre-test and the post-test depending on age—the progress was greater in the 6-year-olds than in the 4-year-olds. This result goes against the assumption that the improvement in task performance after the cognitive training should be greater in younger children due to their more extensive brain plasticity (26) and their neural networks which are much less specialized as the correlates of the trained function in children (27). However, there is a very scarce amount of research on attention training during early and middle childhood (which was one of the reasons for undertaking the present study). The meta-analysis of study results regarding the effectiveness of attention training, prepared by Peng and Miller (24), included only two studies with participants aged 4 or 5 years old (other studies start from the age of 8). It is, therefore, probably true that the effects of cognitive interventions in children, though observable, are limited by the starting (lower) level of the structural development of the brain and cognitive functions, e.g., patterns of synaptic connections or myelination of nerve fibers (30). It would mean that the effects of the executive attention training are limited due to the current stage of the cognitive and neurological development of the children, i.e., they cannot certain capabilities fully or achieve a specific level of task performance if they draw on simpler, yet immature processes or if their neural networks (the correlates of such capabilities) are not fully developed yet (30, 41). The functions of executive attention are complex control functions that depend on the prefrontal cortex maturation degree. The prefrontal and frontal areas develop during middle childhood, and children gradually enhance the ability of alternating attention (35), response inhibition (50), and the performance of tasks that involve a spatial conflict or require the control of interference (51). Such tasks were included in the training application designed for the present study. The 4-year-old participants found these tasks too difficult and were unable to perform them since the required capabilities were still at an early stage of development. Therefore, it is probable that the mechanism of executive attention in four-year-olds is not so much less responsive to training than in 6-year-olds as it has developmental limits due to the lower advancement of the nervous system and, consequently, of the trained functions themselves.

As regards the scope of the WM transfer, the magnitude of the effect for the measurement interaction and the type of the group is not satisfactory, i.e., it is significant, though only medium. It can suggest a limited scope of the near transfer of the executive attention training effects onto an untrained function, i.e., WM (performance of *n*-back tasks).

The most popular models of working memory (52–54) include the central executive component identified with executive attention. Numerous authors (17, 18, 55) assert that the efficiency of the central executive component ensures a greater capacity of WM and influences its performance. Studies cited in specialist publications confirm that persons with a greater working memory capacity are more effective in performing attentional tasks (17, 56). Moreover, working memory (the central executive component) and executive attention activate the same brain structures, i.e., the cingulate and prefrontal cortex (57). Additionally, the specificity of brain development in childhood e.g., the lower specialization of neural networks making the cortical activation patterns caused by the tasks more extensive (25, 27) supported the assumptions that cognitive interventions would lead to a wider transfer of the cognitive training effects in children. It seemed, therefore, that the improvement of executive attention due to the training should enhance the performance of WM-related tasks. So why are the training effects limited? On the one hand, it could be related to the developmental changes of WM in childhood combined with the use of the n-back task, involving a specific aspect of WM, i.e., updating. Authors assert that the intense increase in the ability to refresh (update) occurs between the ages of 4 and 15 years old (58). The participants of the present research were children aged 4 and 6 years old, so they had only started to develop their refresh/update ability. It could be that Jolles and Crone (30) are correct when they postulate that the effects of training, including the scope of transfer onto untrained cognitive processes, are limited by the lower level of the structural development of the brain and cognitive functions. In other words, many authors uphold the view that one cannot improve something that does not function yet or is at an early stage of development. Therefore, the low starting level of WM development in the studied group could explain the limited scope of the transfer of the attention training effects. On the other hand, the WM measurement was only related to the memory of the object's location in space, so it pertained to just one aspect of WM. Furthermore, the paradigm of n-back tasks was far removed from the children's daily experiences and could cause them some difficulty.

The analysis of publications regarding attention training in middle childhood reveals that no researcher has studied the effects of the transfer of training on the performance of n-back tasks. For this reason, we are unable to draw any comparisons with our results. It was only the experiment by Thorell et al. (48), and the experiment by Pozuelos et al. (59) looked into the transfer of the training effects on working memory, but it used other tasks than n-back tasks (the near transfer was not observed in such a scope as it had been expected). In consequence, it is impossible to determine whether our results are standard for this type of cognitive intervention at this age or whether they should be perceived as atypical.

Finally, the scope of the transfer of the executive attention training onto the performance of n-back tasks may also be limited, probably due to the medium improvement in the performance of trained tasks. It would be unreasonable to expect a wide scope of transfer after the executive attention training if the improvement in the attentional task performance was only medium.

The present study also evaluated the significance of age in the effects of near transfer of attention training. Both training groups, i.e., the 4-year-olds and the 6-year-olds, showed a similar improvement in the performance of n-back tasks due to the executive attention training. In this case, the scope of the near transfer is unrelated to the age of the children participating in the cognitive intervention. Based on the previously mentioned neuropsychological knowledge and the ensuing assumption on the relation between age and the effectiveness of executive attention training, it would seem that the scope of transfer should be greater in younger children. However, improving the WM, as in the case of EA, may have its developmental limits. Hence, developmental readiness for WM changes may be greater in older children. This corresponds to the observation that in 6-year-old children, the n-back task performance was improved in both the training and the active control groups. In contrast, in 4-year-olds, such an improvement occurred only in the EA training group. Thus, in 6-year-olds, the improvement in WM seems to be more due to the general readiness of the neurocognitive system for developmental changes. In contrast, in 4-year-olds, it seems to be more strongly related specifically to the attendance of EA training.

The present study also assumed that executive attention training should allow 4- and 6-year-olds to improve their performance of matrix tasks measuring fluid intelligence (the far transfer of the training effects). The obtained results substantiate the far transfer of the executive training effects in both age groups, though the observed impact is not powerful. Rueda et al. (1, 28) concluded that the transfer of the executive attention training effects onto fluid intelligence was expected since both functions (executive attention and fluid intelligence) have common brain correlates (frontal areas of the brain). In turn, authors (19) postulates that attention resources form the foundation for intelligence, as attention controls other cognitive processes and the effective performance of cognitive tasks. Many studies indicate that attentional control is a correlate and a predictor of fluid intelligence (60, 61). The limited effect achieved in the experimental interventions may result (as in the case of the transfer onto WM) from the medium impact of the training on the performance of attentional tasks—only a medium improvement in the achieved EA task difficulty level will probably not generate a strong effect in terms of the far transfer of the training effects. Furthermore, though specialist publications do underline the connection between executive attention and fluid intelligence (18, 60, 61), the performance of tasks measuring the general mental capabilities is influenced by other variables such as visual perception [which explains as many as 20% of result variance in TMK, (43)] and analogical reasoning. It seems, therefore, that after short training of executive attention, one should not expect any spectacular results in terms of transfer, and the actual result can be seen as satisfactory and promising. The presented data also challenge the thesis that fluid intelligence is genetically conditioned and does not come under the impact of socialization or education (1). The obtained research results support the increasingly frequent assumption that relevant cognitive interventions can improve fluid intelligence at any age (1, 28, 39). However, the conditions and the scope of this improvement remain open questions.

A greater improvement in results was observed in the 4-year-olds than in the 6-year-olds. Similar results were obtained by Rueda et al. (28)—their research also recorded the far transfer of the training effects onto the performance of matrix tasks (results in the K-BIT test, in the fluid intelligence section) and the children at the age of 4 years old showed a greater change in the final test results as compared with the initial measurement than in the case of the 6-year-olds participating in the experiment. Furthermore, the analyses revealed that the 4-year-olds improved the TMK results only in the training group. In turn, the 6-year-olds improved in each group, but the increment in the 6-year-old training group was significantly higher than in the active and passive control groups. The improvement in the results of each group comprising the elder children may, in part, have resulted from the learning effect, as the same tool was used in both measurements (initial and final) of fluid intelligence by the lack of the different test versions in Poland. At the same time, this may again indicate that 6-year-old children are generally more ready for developmental changes, while the specific effect of EA training is greater in 4-year-olds.

The presented results of our research corroborate the effectiveness of the EA training. The performed training was relatively short, i.e., 14 sessions, each lasting around 7–8 min. However, this duration of the training turned out to be sufficient for observing the results presented above. The obtained results may also support the often-accepted assumption that attention is the hub or the core of other cognitive processes and capabilities. The effects of executive attention training extend to working memory and fluid intelligence—if executive attention was not an essential component, the transfer would not be possible (62). Moreover, the results suggest that older children show greater general neurocognitive readiness for developmental changes, while the transfer mechanisms of training effects seem to be stronger in younger children.

## Limitations and perspectives of the future research

The present research is not free from limitations. The pre-test and the post-test used in the experimental procedure lacked other tasks and tools to measure executive attention (e.g., the Child ANT or tasks based on the stop-signal paradigm or sorting of cards). If any improvement in their performance were observed, it might support attention enhancement through executive attention training and not only improve the performance of trained tasks. Also, regarding the WM measurement, the use of tasks other than n-back tasks (which pertained only to the memory of the stimulus location in space in the present study) and the improvement in their performance after the training would suggest the enhancement of WM in general. Furthermore, including other tasks measuring various cognitive functions and analyzing the scope of improvement in their performance could have answered the question regarding the mutual dependencies between cognitive processes in children and the correlates (or even predictors) of the scope of transfer.

The present research lacks a follow-up evaluation, so it is impossible to determine the durability of the intervention's effects.

The presented results are averaged results for the specific groups of children. The extreme diversity of the measurement results of all variables, both in the pre-test and the post-test, is evidenced by the magnitudes of the confidence intervals. This method of analysis ignored the intra-subject variability. However, the analyses could not use models that account for the inter- and intra-subject variability, e.g., LGCM models, due to the small size of the groups, different time intervals between the training sessions, and varied trajectories of changes resulting from the training for particular children (63).

Despite the introduction of randomization, it was not possible to balance the groups (especially the 6-year-old children groups) in terms of the baseline level of the cognitive functions studied. This is a potential confounding variable that may impact the training outcomes.

It is difficult to make broad generalizations based on the obtained results. One may only apply them to children aged 4 and 6 attending urban kindergartens. Future research should extend the studied groups to include children from other age groups and children attending rural kindergartens, etc., to make the sample more representative.

The authors who study attention training in children [e.g., (64)] emphasize that it is impossible to determine how the improvement in the tasks performed in experimental conditions and using standardizes tools outside the real-life environment translates into the daily functioning of the research participants. At the same time, the researchers postulate that future studies should include the aspect of the environmental diagnosis (e.g. observations of the child's behaviors in kindergarten/school, school grades, assessment of the learning effectiveness etc.) before and after the training. It would allow us to evaluate the usefulness of cognitive training as a beneficial intervention from both the practical and the scientific perspectives. Therefore, future research should include this dimension.

The study also did not include variables such as the education level of the parents of the children studied or the family's socioeconomic status. The analysis could include this variable as a control or covariate variable.

## Data Availability

The raw data supporting the conclusions of this article will be made available by the authors, without undue reservation.
